# A perturbation approach for refining Boolean models of cell cycle regulation

**DOI:** 10.1371/journal.pone.0306523

**Published:** 2024-09-06

**Authors:** Anand Banerjee, Asif Iqbal Rahaman, Alok Mehandale, Pavel Kraikivski

**Affiliations:** 1 Division of Systems Biology, Academy of Integrated Science, Virginia Polytechnic Institute and State University, Blacksburg, VA, United States of America; 2 VT-Center for the Mathematics of Biosystems, Virginia Polytechnic Institute and State University, Blacksburg, VA, United States of America; 3 Department of Computer Science, Virginia Polytechnic Institute and State University, Blacksburg, VA, United States of America; Universidad Adolfo Ibanez, CHILE

## Abstract

Considerable effort is required to build mathematical models of large protein regulatory networks. Utilizing computational algorithms that guide model development can significantly streamline the process and enhance the reliability of the resulting models. In this article, we present a perturbation approach for developing data-centric Boolean models of cell cycle regulation. To evaluate networks, we assign a score based on their steady states and the dynamical trajectories corresponding to the initial conditions. Then, perturbation analysis is used to find new networks with lower scores, in which dynamical trajectories traverse through the correct cell cycle path with high frequency. We apply this method to refine Boolean models of cell cycle regulation in budding yeast and mammalian cells.

## Introduction

The cell cycle is the complex process through which a cell grows and divides into two genetically identical daughter cells. The progression of a cell through the cell cycle can be viewed as a trajectory through a multidimensional space of protein activation states which is controlled by an intricate network of biochemical reactions. The reaction network is fundamentally identical in all eukaryotes, and involves the activation/deactivation of different Cyclin-Cdk complexes at different stages of the cell cycle.

Most mathematical models of cell cycle are Ordinary Differential Equation (ODE) based [1–6]. While these models provide complete information about the time evolution of concentrations of different molecular species, they often require a large number of parameters in the form of reaction rate constants, whose values are often not known.

In studies of biological processes where detailed temporal information is not necessary, Boolean modeling can be used to analyze qualitative behavior of the system [7]. This approach eliminates the need for kinetic parameters. The model consists of a network with nodes and edges. The nodes represent genes or proteins, and the edges signify reaction connecting them. Gene/protein activity is often represented by binary values, 1 and 0, denoting active and inactive states, respectively. The activity values are updated using logic-based rules. Boolean modeling has been used to study a wide range of biological phenomena, including gene regulatory networks [8], signaling pathways [9], cell fate decisions [10], and cell cycle [11–14].

The dynamics of a Boolean network depend not only on its update rules but also on the order in which these updates occur, commonly referred to as the update scheme [15]. In general, the update scheme can be defined as synchronous or asynchronous. In the synchronous update scheme, the values of all variables are updated simultaneously in one update step. The synchronous update is computationally easy to implement; however, it can produce spurious cycles in the dynamics. In contrast, in asynchronous update schemes, a randomly chosen variable is updated in each time step, resulting in stochastic representation of the dynamics. In this method, the same reaction can occur multiple times before the next reaction occurs, allowing simulation of biological processes that involve reactions on slow and fast time scales. A drawback of the asynchronous scheme is that the run time for simulations increases rapidly with the number of nodes in the network [16].

Among the Boolean models of cell cycle, Li et al. [11] studied the cell cycle network in budding yeast using synchronous update scheme, and found that the steady state with the largest basin of attraction is stable to perturbations, and the correct sequence of protein activation states during cell cycle, is the globally attracting trajectory of the dynamics. Davidich et al. [12] found similar results in the case of cell cycle networks in fission yeast. Faure et al. [13] used both synchronous and asynchronous update schemes to analyze mammalian cell cycle networks, and found that synchronous update resulted in two attractors: a steady state corresponding to G0 (with the inhibitors of cell cycle active), and a cyclic attractor corresponding to the cell cycle. Asynchronous update preserved the G0 steady state, but the cyclic attractor observed in the case of synchronous update scheme changed to a complex attractor made up of many intertwined cycles.

Although these studies have laid the groundwork, they include only a small subset of the genes/proteins involved in the cell cycle. Furthermore, the models were developed in a user-centric manner as opposed to a data-centric manner. To develop more comprehensive data-centric models, a systematic and automated approach is needed to construct the initial network and then incorporate additional nodes into a growing network.

**Fig 1:**
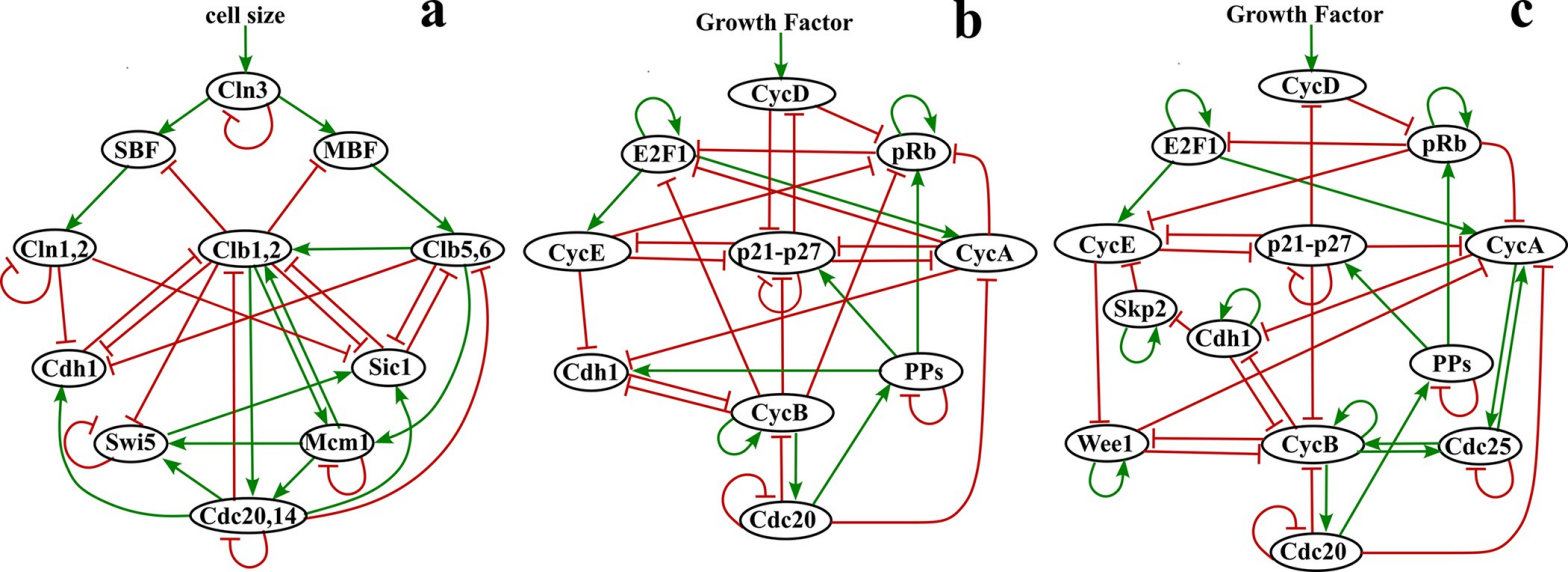
Three cell cycle models. (a) The budding yeast cell cycle regulation network derived from Li et al., model [11]. (b), (c) Mammalian cell cycle regulation networks derived from Novak-Tyson and Gerard-Goldbeter models [1, 2], respectively. Oval shapes (network nodes) are proteins and lines between proteins (directed edges) are interactions that are of two types: activation represented by green arrow-headed lines and inhibition represented by red bar-headed lines.

In this study, our aim was to develop a method for refining Boolean models of cell cycle so that the model dynamics follows the correct cell cycle trajectory with high frequency. To achieve this goal, we analyzed three cell cycle models shown in Fig 1: (a) Model A: the budding yeast cell cycle model [11], (b) Model B: the mammalian cell cycle regulation derived from Novak-Tyson model [1] and, (c) Model C: the mammalian cell cycle control derived from Gerard-Goldbeter model [2]. We defined a network score which quantifies the consistency of the model dynamics with that of the cell cycle. All single-edge and double-edge perturbations of the networks were analyzed to search for new interactions that improve the network score. For each model, we found new interactions that improve the network score. We compared those interactions with the ones listed in the protein-protein interaction database SIGNOR 3.0 [17]. By filtering out the interactions that are inconsistent with the database, we were able to produce refined versions of all the three models under study. Our future plan is to is to enhance the level of automation in our analysis and develop approaches to handle larger number of nodes and edges in the network.

## Results

### Budding yeast: Model A

To test and optimize our perturbation-based approach, we first analyzed the interaction network controlling the cell cycle in budding yeast, shown in Fig 1A. The network was taken from Li et al. [11]. As mentioned previously, Li et al., used synchronous update scheme and analyzed the robustness of the network by calculating the effect of single perturbations on the size of the largest attractor. We furthered that analysis by using the asynchronous update method, and a more comprehensive scoring scheme to quantify perturbations–one that includes the basin sizes of all the steady states, as well as the sequence of protein activation events. (See Methods for the definition of network score)

The network in Fig 1A has 11 nodes, resulting in 2^11^ = 2048 initial conditions. All initial conditions eventually reach one of the 7 steady states listed in Table 1. As expected, these 7 steady states are same as those found in Ref. Li et al. [11], but due to the asynchronous updating rule used in our simulations, the size of the basin of attraction corresponding to each steady state is slightly different in comparison. The steady state with the largest basin of attraction (in bold) corresponds to the G0 phase of the cell cycle, in which the inhibitors of the cell cycle Cdh1 and Sic1 are active, and the cell is in a resting phase (neither dividing nor preparing to divide).

**Table 1:** List of steady states and the corresponding sizes of basin of attraction. The basin sizes were obtained using asynchronous update scheme.

Cln3	MBF	SBF	Cln1,2	Cdh1	Swi5	Cdc20,14	Clb5,6	Sic1	Clb1,2	Mcm1,SFF	Basin size
**0**	**0**	**0**	**0**	**1**	**0**	**0**	**0**	**1**	**0**	**0**	**1151**
0	0	0	0	0	0	0	0	1	0	0	374
0	0	1	1	0	0	0	0	0	0	0	257
0	1	0	0	1	0	0	0	1	0	0	155
0	1	0	0	0	0	0	0	1	0	0	48
0	0	0	0	0	0	0	0	0	0	0	37
0	0	0	0	1	0	0	0	0	0	0	26

**Fig 2:**
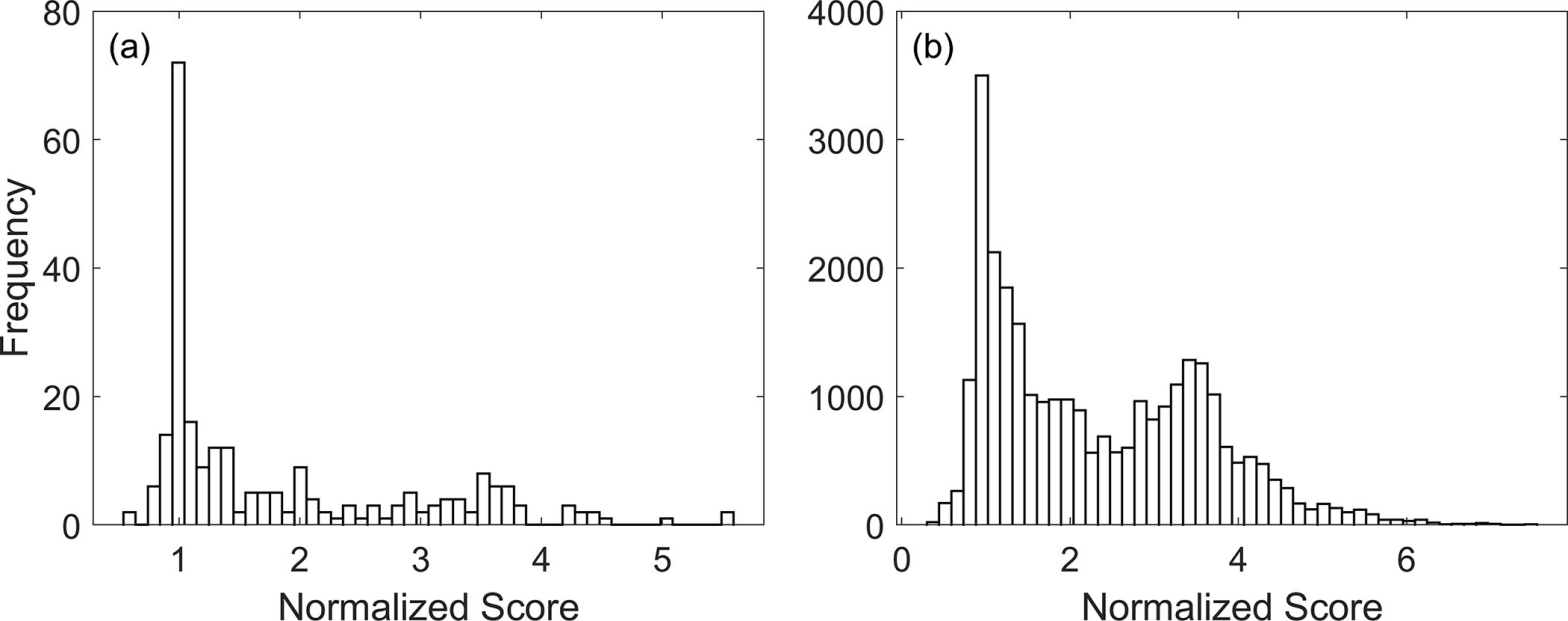
Model A: Histogram of normalized network scores after single-edge perturbation (a) and double-edge perturbation (b). The normalized scores were calculated by dividing the scores of the perturbed networks with the score of the original network. 28% of the single-edge and 14% of the double-edge perturbations had a normalized score less than one.

Next, we introduced single-edge and double-edge perturbations (see Methods for the definitions) to the network, aiming to identify perturbations that decrease the network score. Fig 2 shows the histogram of normalized scores after single-edge and double-edge perturbations of the network. Notably, in both instances, the distribution’s peak aligns with the score of the original network. However, intriguingly, a significant proportion of the single-edge (28% of the total) and double-edge perturbations (14% of the total) led to a lower network score, i.e., a normalized score less than one. The complete list of network scores corresponding to single-edge and double-edge perturbations is given in the S1 Table.

From the perturbations resulting in a score smaller than the original graph, the ones which are consistent with experimental data are listed in Table 2. For example, Skotheim et al., observed that Cln1/2 dependent positive feedback promotes coherent SBF and MBF regulated gene expression [15]. Therefore, the addition of a positive influence of Cln1/2 on MBF can improve the consistency of the modeled cell cycle regulation with data. For the other two interactions in Table 2, it is well known that the transcription factor SBF controls CLN1/2 transcription, and the transcription factor MBF regulates CLB5/6 [18, 19], some data also suggest that in the absence of MBF, SBF is able to regulate expression of Clb5 [20]. Also, deletion analysis of upstream DNA sequences shows that Cln2 transcription can be induced by MBF transcription factor [21]. Furthermore, some known cell cycle models incorporate the positive regulation from SBF on Clb5,6 and MBF on Cln1,2 in addition to SBF→Cln1,2 and MBF→Clb5,6 [4, 22]. These additional interactions allowed the models to correctly characterize a vast amount of gene deletion mutant strains and better explain the regulation of cell cycle START transition. Further, removing a direct regulation of Mcm1 transcription factor by Clb5/6 is another way to improve the network score. This interaction has been introduced by Li et al. [11] to simplify the original cell-cycle regulation from Chen et al. [3] by removing DNA replication event that is controlled by Clb5/6. Thus, the control of DNA replication by Clb5/6 was bypassed by the direct Cln5/6→Mcm1 interaction that drives cell cycle into G2-M phases. Therefore, the direct activation of Mcm1 by Clb5/6 can be removed since this regulation is not supported by experimental data.

**Table 2:** List of single-edge and double-edge perturbations of the network in Model A that are consistent with experimental data and result in a network score smaller than the original network. The last three columns show the percentage of trajectories that go through the correct sequence of protein activation states (correct), incorrect sequence of protein activation states (incorrect), and return back to G0 state without ever reaching the S phase of the cell cycle (did-not-start).

Perturbation	Normalized Network Score	steady state count	Largest basin size	Correct	Incorrect	Did-not-start
**Original Graph**	**1**	**7**	**1151**	**42%**	**16%**	**42%**
Single-edge perturbation						
Cln1,2-to-MBF: 0 to 1	0.59	6	1322	55%	20%	25%
MBF-to-Cln1,2: 0 to 1	0.85	5	1296	54%	21%	25%
SBF-to-Clb5,6: 0 to 1	0.87	7	1280	46.5%	28%	25.5%
Clb5,6-to-Mcm1: 1 to 0	0.88	7	1161	58%	0%	42%
Double-edge perturbation						
Cln1,2-to-MBF: 0 to 1 Clb5,6-to-Mcm1: 1 to 0	0.44	6	1344	74%	0%	26%
Cln1,2-to-MBF: 0 to 1 Cdh1 to Swi5: 0 to -1	0.61	6	1317	52%	19%	29%
MBF-to-Cln1,2: 0 to 1 SBF-to-Clb5,6: 0 to 1	0.71	5	1420	59%	34%	7%

Interestingly, double perturbation Cln1,2-to-MBF: 0 to 1 and Clb5,6-to-Mcm1: 1 to 0 resulted in the largest drop in the network score. The combination of these perturbations increased the basin size of the largest steady state as well as the frequency with which the dynamics follows the correct trajectory. From the network it is clear that positive regulation of transcription factor MBF by Cln1,2 improves the chances of Clb5,6 activation, which marks the point-of-no-return for this model. This results in more trajectories going through the cell cycle. We also found that the non-biological steady state [Cln3 = 0, MBF = 0, SBF = 1, Cln1,2 = 1, Cdh1 = 0, Swi5 = 0, Cdc2014 = 0, Clb5,6 = 0, Sic1 = 0, Clb1,2 = 0, Mcm1,SFF = 0] is absent in the presence of this double perturbation.

### Mammalian cell: Model B

Next, we analyzed the ODE-based Tyson-Novak model [1] of cell cycle in mammalian cells. Guided by the ODEs, we deduced the molecular interaction network (Fig 1B) that we analyzed using the Boolean approach. Our model has 10 nodes, resulting in 2^10^ = 1024 initial conditions. We found the largest attractor was the G0 state with a basin of attraction of size 283. The complete list of steady states (98 in total) and the corresponding basin sizes are given in the S2 Table.

**Fig 3:**
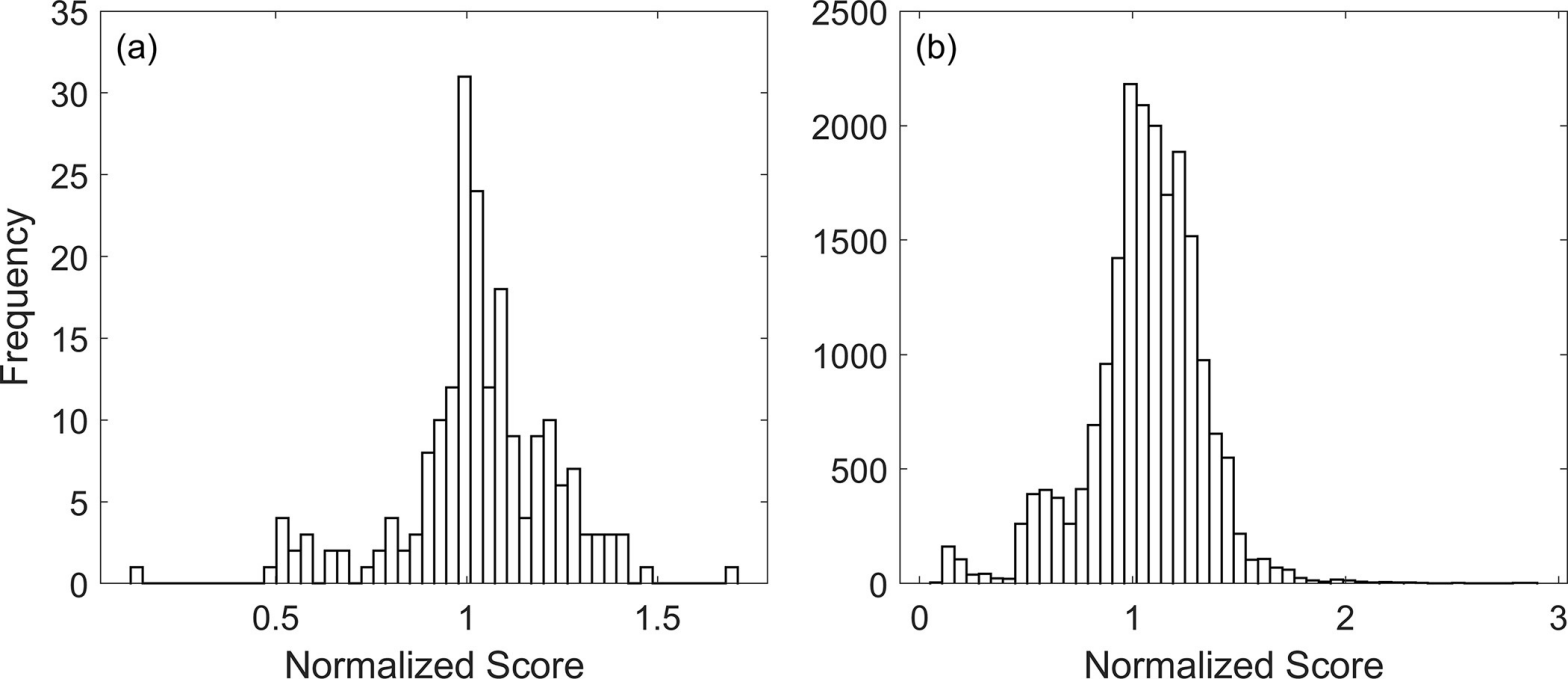
Model B: Histogram of normalized network scores after single-edge perturbation (a) and double-edge perturbation (b). The normalized scores were calculated by dividing the scores of perturbed networks with the score of the original network. 39.5% of the single-edge and 35.2% of the double-edge perturbations had a normalized score less than one.

Like previously, we used the perturbation analysis to find networks with a lower score. The distributions of scores after single-edge and double-edge perturbations are shown in Fig 3. The distribution again peaks at the original graph score, but in this case, we find more perturbations– 39.5% of single-edge and 35.2% of double-edge—resulting in a lower graph score. The full list of network scores corresponding to single-edge and double-edge perturbations is given in the S2 Table.

In Table 3 we list the perturbations that are consistent with experimental data and also result in a score lower than the score computed for the original network. In this case, the validity of perturbations were determined by comparing them against known protein-protein interaction listed in the database SIGNOR 3.0 [17] (see S1 Text for details).

**Table 3:** List of single-edge and double-edge perturbations of the network in Model B that are consistent with experimental data and result in a network score smaller than the original network. The last three columns show the percentage of trajectories that go through the correct sequence of protein activation states (correct), incorrect sequence of protein activation states (incorrect), and return back to G0 state without ever reaching the S phase of the cell cycle (did-not-start).

Perturbation	Normalized Network Score	Steady state count	Largest basin size	Correct	Incorrect	Did-not-start
**Original Graph**	**1**	**98**	**283**	**58%**	**0%**	**42%**
Single-edge perturbation						
CycE-to-CycE: 0 to -1	0.5	167	640	53%	3%	44%
CycA-to-CycE: 0 to -1	0.52	219	640	55%	0%	45%
pRB-to-CycE: 0 to -1	0.65	34	640	50%	2%	48%
Double-edge perturbation						
CycE-to-CycE: 0 to -1 CycA-to-CycE: 0 to -1	0.58	171	633	59%	4%	37%

The single-edge perturbations CycE-to-CycE: 0 to -1 and CycA-to-CycE: 0 to -1 cause large drops in the network score. The effect of both perturbations is to turn off the CycE activity after the cell cycle has entered the S phase. The interaction corresponding to the perturbation CycA-to-CycE: 0 to -1, namely, negative autoregulation of CycE, was indeed observed in Ref. [23], where autophosphorylation of CycE-Cdk2 complex results in ubiquitin-mediated degradation of CycE. The interaction corresponding to the perturbation CycA-to-CycE: 0 to -1, namely negative regulation of CycE by CycA does not exist in databases. However, the known sequence of interactions CycA ⊣ Cdh1 ⊣ Skp2 ⊣ CycE results in the indirect negative regulation of CycE by CycA. Since Skp2 is not a part of Model B, we think the perturbation CycA-to-CycE: 0 to -1 tries to capture the indirect negative interaction with a direct one. Interestingly, the perturbation pRB-to-CycE: 0 to -1 is also present in our network for the Model C (Fig 1C). Further, Model B has a lower graph score when the negative regulation of Cdh1 by CycE is removed from the network. This interaction is not found in databases and also not included in Model C (Fig 1C).

When both network edges CycE-to-CycE: 0 to -1 and CycA-to-CycE: 0 to -1 in Model B are simultaneously modified the graph score drops a little bit lower compared with the score results for corresponding single-edge perturbations. The graph score is also better when the activation of p27 by phosphatases PPs is removed and self-activation loop to Cdh1 is added. The last regulation is also present in Model C (Fig 1C). Overall, almost all interaction modifications that give lower graph scores for Model B in our perturbation analysis are already present in Model C that we analyze next.

### Mammalian cell: Model C

Finally, we constructed a Boolean model using the ODE-based Goldbeter model of mammalian cell cycle [2]. The network of interaction is shown in Fig 1C. Compared to Model B in Fig 1B, this network has additional nodes Skp2, Wee1, and Cdc25. For this model we find that the dynamics for all initial conditions converge to the attractor corresponding to G0 state listed in Table 4.

**Table 4:** Steady state and the basin size for the Goldbeter model.

CycD	CycE	CycA	CycB	E2F	Skp2	Cdh1	Cdc25	pRB	p21-p27	Cdc20	Wee1	PPs	Basin size
0	0	0	0	0	0	1	0	1	0	0	1	0	8192

**Fig 4:**
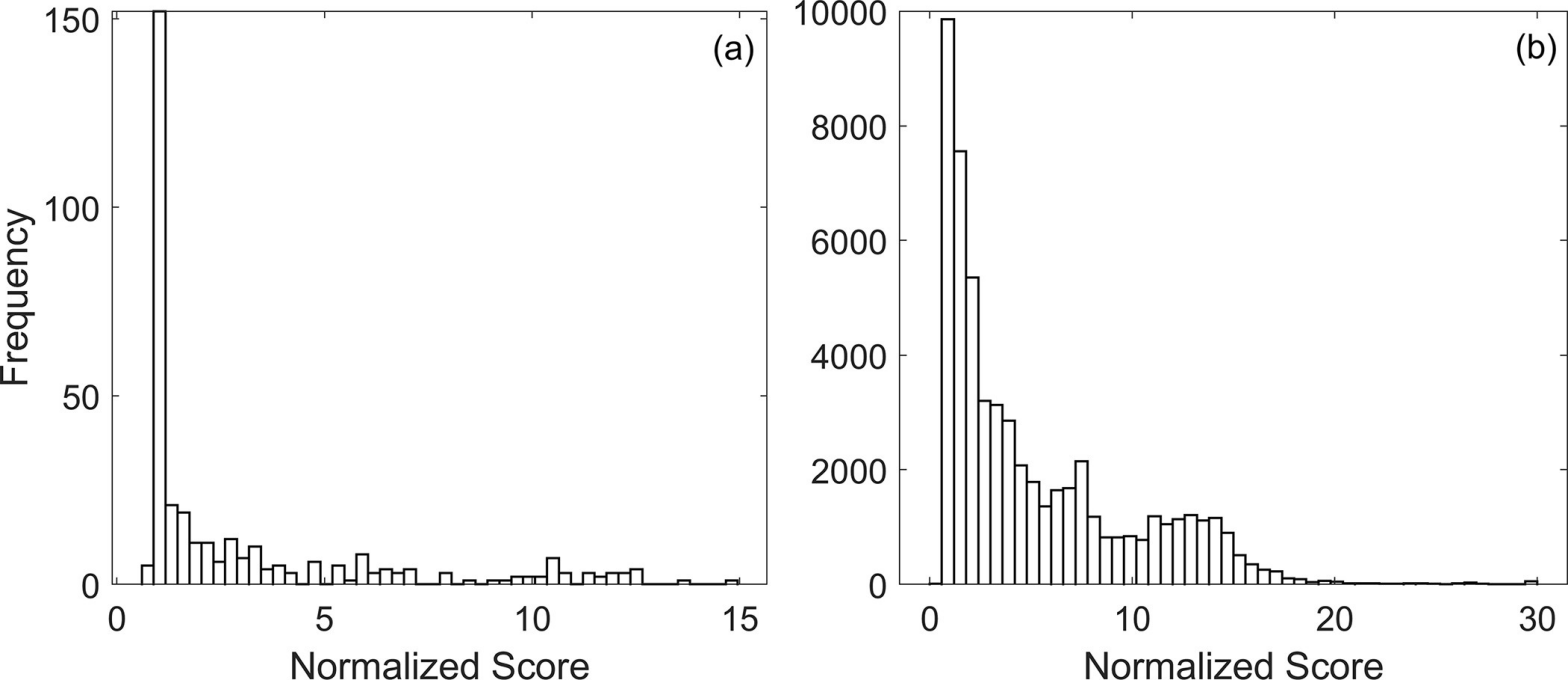
Histogram of normalized network scores after single perturbation (a) and double perturbation (b). The normalized scores were calculated by dividing the network scores with the score of the original network. 35.2% of the single-edge and 2% of the double-edge perturbations had a normalized score less than one.

The distribution of scores after single-edge and double-edge perturbations are shown in Fig 4. The original network gives excellent results, and as a consequence, relatively few perturbations resulted in a lower score. The complete list of network scores corresponding to single-edge and double-edge perturbations is given in the S3 Table.

The perturbations that are consistent with experimental data and also result in a score lower than the score for the original network are listed in Table 5. The double-edge perturbation CycA-to-CycE: 0 to -1 and RB-to-RB: 1 to 0, resulted in the lowest graph score. Remarkably, the indirect negative regulation of CycE by CycA (CycA ⊣ E2F1 → CycE) is present in Model B. This double-edge perturbation improved the frequency of starting the cell cycle as well as the frequency of correct trajectories. Interestingly, the single perturbation RB-to-RB: 1 to 0 has a score of 13.8, but when combined with CycA-to-CycE: 0 to -1, results in a score of 0.85. The second lowest graph score was obtained by adding a negative interaction from phosphatases PPs on Skp2 and a negative regulation of p21 and p27 by Skp2. Both perturbations are found in databases. For example, dephosphorylation of Skp2 by the mitotic phosphatase Cdc14B promotes the degradation of Skp2 [24]. Also, Skp2 is required for ubiquitin-mediated degradation of p27 and p21 [25, 26]. The double-edge perturbation Cdc25-to-CycE: 0 to 1 and Cdh1-to-Cdc25: 0 to -1 also improves the graph score. The up-regulation of CycE by Cdc25A has been confirmed in Ref. [27]. It has been also observed that Cdc25A degradation is mediated by the anaphase-promoting complex (APC/C)(Cdh1) [28]. The final suggestion in the list of Table 5 is pRB-to-CycA: -1 to 0 and PPs-to-Wee1: 0 to 1 double-edge perturbation. The positive regulation of Wee1 by phosphatases agrees with studies reporting that phosphatase Cdc14A inhibits Wee1 degradation through dephosphorylation [29]. Also, the direct negative regulation of CycA by pRB does not appear in databases. Further, Model C has already a negative indirect influence of pRB on CycA (pRB ⊣ E2F1 → CycA). Thus, the direct inhibition of CycA by pRB is redundant.

**Table 5:** List of single-edge and double-edge perturbations of the network in Model C that are consistent with experimental data and result in a network score smaller than the original network. The last three columns show the percentage of trajectories that go through the correct sequence of protein activation states (correct), incorrect sequence of protein activation states (incorrect), and return back to G0 state without ever reaching the S phase of the cell cycle (did-not-start).

Perturbation	Normalized Network Score	Steady state count	Largest basin size	Correct	Incorrect	Did-not-start
**Original Graph**	**1**	**1**	**8192**	**54%**	**2%**	**44%**
Single Perturbation						
CycA-to-CycE: 0 to -1	1	1	8192	54%	1%	45%
CycE-to-CycA: 0 to 1	0.98	1	8192	55%	2%	43%
CycA-to-p21-p27: 0 to -1	0.98	1	8192	55%	2%	43%
pRB-to-CycA: -1 to 0	0.98	1	8192	55%	2%	43%
Double Perturbation						
CycA-to-CycE: 0 to -1 pRB-to-pRB: 1 to 0	0.85	1	8192	61%	0%	39%
PPs-to-Skp2: 0 to -1 Skp2-to-p21-p27: 0 to -1	0.9	1	8192	58%	3%	39%
Cdc25-to-CycE: 0 to 1 Cdh1-to-Cdc25: 0 to-1	0.94	1	8192	57%	7%	36%
pRB-to-CycA: -1 to 0 PPs-to-Wee1: 0 to 1	0.96	1	8192	56%	2%	42%

## Discussion

Understanding the complex dynamics of the cell cycle is of both theoretical and practical interest. Considerable effort has been put into developing ODE-based and Boolean dynamical models of cell cycle, that are consistent with experimental data. The main challenge in developing such dynamical models is the lack of a systematic approach in putting together the model, and later incorporating new genes and proteins in the model as new information becomes available. The lack of such a structured approach is apparent among existing cell cycle models, which exhibit differences in their sets of variables and the interaction networks connecting them.

In this manuscript, we developed a semi-automated method to construct and improve Boolean models of cell cycle. The main steps in our approach are as follows: (a) manually construct an initial Boolean model using existing Boolean or ODE models of the cell cycle (b) define a network score to quantify the consistency of network dynamics with that of the cell cycle, (c) using single-edge and double-edge perturbations of the network, find interactions that result in a lower network score, and (d) compare the proposed interactions in (c) with protein-protein interaction databases to find those that are consistent with experimental data. Edge perturbations have been previously used in several studies, including the study different classes of mutations in human inherited disorders [30], genotype-phenotype associations using alleles that lack single interactions in C. elegans [31], removal of unwanted attractors such as the cancerous attractor in T-LGL leukemia [32], and the stability of the largest attractor in budding yeast cell cycle [11]. Our goal, however, was to develop a perturbation technique that allows us to refine cell cycle models, ensuring that the refined models exhibit correct cell cycle dynamics. Importantly, our approach should also facilitate automation of the model refinement process.

We first applied our perturbation method to the well-known model of cell cycle in budding yeast. Through double perturbation analysis, we found interactions that significantly improved the basin size of the largest attractor and the frequency of transitions through the correct cell cycle trajectory. Interestingly, these interactions (Cln1,2-to-MBF: 0 to 1, Clb5,6-to-Mcm1: 1 to 0) are not present in any database, but since they emerged naturally from our analysis, searching for them in the literature became much easier. We then applied the same method to two different models of cell cycle in mammalian cells: the Tyson-Novak and Goldbeter model. In both cases, we found perturbations that improved the network score. Thus, our approach resulted in the refinement of all three models that we studied.

Currently, the main limitation of our approach is that after selecting the interactions that are consistent with the data available in the SIGNOR database, we still need to manually accept or reject them. This is because the SIGNOR database is not complete, and sometimes the format in which the information is stored does not allow us to uniquely identify the interaction nodes. For example, CDK2 in the SIGNOR database corresponds to both Cyclin E and Cyclin A. Nevertheless, as the quality of databases improves, the need for manual intervention will also go away. The best-case scenario will be that all interactions with a lower network score and consistent with the database are incorporated to create a new refined model.

Another aspect of developing data-centric models needing attention is an automated method for adding more cell cycle genes to the existing network. This would result in more detailed models that can be used to study gene knockout experiments. As a preliminary step, we explored adding one new node at a time, selecting the gene with the highest connectivity (number of interactions) to the existing nodes in the network. We searched through the SIGNOR database and identified MYC, CHECK1, and TP53 as the new genes that should be added to the network, one gene at a time. These genes play important roles in the cell cycle and would be reasonable additions. Thus, this simple approach provides an automated method of adding new genes to the existing network. We envision that the iterative process of network growth and refinement will provide the means for developing data-centric Boolean models of cell cycle.

The number of double-edge perturbations increases as N^4^ with the number of nodes in the network. Combining this with the fact that for each network perturbation multiple simulation runs are needed to compute the statistical properties of the dynamics, the computation time increases significantly with the number of nodes. However, we found that retaining only the feasible single perturbations for the double perturbation analysis, significantly reduced the computational burden. For example, for Model C, about 80% of all single perturbations were classified as ‘false’ after database search, and retaining only the ‘true’ single perturbations for the double perturbation analysis reduced the search to only about 4% of all possible double perturbations.

## Methods

### Cell-cycle models and update scheme

The Boolean models of cell cycle contain nodes and directed edges. The nodes represent proteins and the directed edges correspond to the interactions between them. The edges are of two different types, inhibitors (-1 weight) and activators (+1 weight). The edges can also be self-loops, i.e. directed edges starting and ending at the same node. The state of the system is described by an *N*-component vector (*X*_1_, *X*_2_,…,*X*_*N*_), where *N* is the number of nodes in the network and *X*_*k*_ describes the activity of the protein corresponding to k-th node in the network; *X*_*k*_ = 1 if the k-th node is active and *X*_*k*_ = 0 if it is inactive.

We use the asynchronous update scheme, in which each node is equally likely to be updated at any time step, to calculate the network dynamics. At each time step, a node is selected randomly and its value is updated using the following rule

$$X_{i}(t+1)=\left\{ \begin{array}{l} 1\;\mathrm{if}\;\sum_{j}a_{ij}X_{i}(t)>0\\ 0\;\mathrm{if}\;\sum_{j}a_{ij}X_{i}(t)<0\\ X_{i}(t)\;\mathrm{if}\;\sum_{j}a_{ij}X_{i}(t)=0 \end{array}\right.$$ (1)

Here *a*_*ij*_ are the weights of the edges joining node *i* to node *j*. We chose *a*_*ij*_ = 1 for activation and *a*_*ij*_ = −1 for inhibition. Since an asynchronous update scheme results in stochastic trajectories, we simulated multiple trajectories for each initial condition to obtain the statistical details of the model dynamics. We follow the same update scheme throughout the publication, except where explicitly mentioned.

### Classification of trajectories

For each model, we fix a protein whose activation is considered as the start of the cell cycle. We call the activation of this protein as the point-of-no-return. If in a simulation the protein is not activated and the trajectory reaches the steady state corresponding to G0, we classify the trajectory as ‘did-not-start’. If the dynamics passes the point-of-no-return, we check for a predefined order of protein activation to classify the dynamics as correct or incorrect. If the dynamics progresses via the predefined sequence of states, we classify the trajectory as ‘correct’, and otherwise ‘incorrect’. Examples of each type of trajectory is given in the SI, and the point of no return, G1 states, and the correct sequence for different models are given below.

Model A (Budding Yeast Model)

In the network shown in Fig 1A, the cell size acts as the start signal. In the absence of this signal the cell remains in G0 phase (a steady state of the model), with the inhibitors of the cell cycle Cdh1 and Sic1 being active. In the presence of the signal the cell enters the G1 phase where Cln3 is activated. At this point the cell cycle can move forward to the S phase by activating Clb5,6 or go back to the G0 phase (we refer to this as the did-not-start case). Upon Clb5,6 activation the cell is committed to go through the cell cycle. The cycle is characterized by the sequential activation of Cln3, Cln1,2, Clb5,6, and Clb1,2.

Point of no return: Clb5,6 activation

G1 states: [Cln3, Cdh1, Sic1] = 1 and [Cdc20,14, Clb5,6, Cln1,2, Clb1,2, Mcm1, SFF] = 0.

Correct sequence: [Clb5,6 = 1, Clb1,2 = 0, Cdc20,14 = 0] → [Clb1,2 = 1, Cdc20,14 = 0] → [Cdc20,14 = 1]

Model B (Modified Tyson-Novak Mammal Model)

Point of no return: CycE activation

G1 states: [CycD, RB, P27, Cdh1] = 1 and [CycE, CycA, CycB, Cdc20] = 0.

Correct sequence: [E2F = 1, CycE = 0, CycA = 0, CycB = 0, Cdc20 = 0] → [CycE = 1, CycA = 0, CycB = 0, Cdc20 = 0] → [CycA = 1, CycB = 0, Cdc20 = 0] → [CycB = 1, Cdc20 = 0] → [Cdc20 = 1]

Model C (Modified Goldbeter Mammal Model)

Point of no return: CycE activation

G1 states: [CycD, RB, Wee1] = 1 and [CycE, CycA, CycB, E2F, Cdc20] = 0.

Correct sequence: [E2F = 1, CycE = 0, CycA = 0, CycB = 0, Cdc20 = 0] → [CycE = 1, CycA = 0, CycB = 0, Cdc20 = 0] → [CycA = 1, CycB = 0, Cdc20 = 0] → [CycB = 1, Cdc20 = 0] → [Cdc20 = 1]

### Perturbations and scoring

We introduced single-edge perturbations in the network by altering an edge value according to the options outlined in Table 6.

**Table 6:** Description of single-edge perturbations.

Perturbation	Description
0 → -1	Addition of an inhibiting edge
0 → 1	Addition of an activating edge
1 → 0	Activating edge replaced with no edge
1 → -1	Activating edge replaced with inhibiting edge
-1 → 0	Inhibiting edge replaced with no edge
-1 → 1	Inhibiting edge replaced with activating edge

We analyzed all possible perturbation to all edges (including self-loops), resulting in a total of 2*N*^2^ single-edge perturbations. Double-edge perturbations were introduced by applying single-edge perturbation to two different edges at a time.

The perturbation analysis was designed to search for networks in which the model dynamics follows the correct cell cycle trajectory with high frequency. Since the trajectories follow stochastic dynamics, for each network we observed trajectories that fall into the groups ‘correct’, ‘incorrect’, and ‘did-not-start’. Using the frequency with which each type of trajectory was observed, we defined a network score to quantify the performance of perturbed networks. The network score is defined as

$$network\;score=\sum_{n=1}^{2^{N}}S({ic}_{n})+\frac{2^{N}}{N_{G1}}\sum_{n=1}^{N_{G1}}Y({ic}_{n})+\frac{2^{N}}{N_{G1}}\sum_{n=1}^{N_{G1}}Z({ic}_{n}),$$ (2)

where

$$S({ic}_{n})=\sum_{k=1}^{N}\left|X_{k}^{ss}({ic}_{n})-{G0}_{k}\right|,$$ (3)

The first term in the network score is the penalty associated with initial condition not reaching the G0 state. It is determined by summing *S*(ic_*n*_): the element-wise difference between the G0 state and the steady state corresponding initial conditions over all 2^*N*^ initial conditions. In Eq 3, $X_{k}^{ss}({ic}_{n})$ is the *k*^*th*^ component of the steady state corresponding to the initial condition ic_*n*_, and G0_*k*_ is the *k*^*th*^ component of the G0 state. In the second term in the network score, *Y*, is for the sequence-related penalty; it takes the value 1 if the stochastic trajectory is classified as ‘incorrect’ and 0 otherwise. The quantity *Z* is the penalty associated with the ‘did-not-start’ case. It takes the value 1 if the trajectory did-not-start and 0 otherwise. The ‘incorrect’ and did-not-start related penalties were introduced only for the G1 states because we were interested in biologically meaningful trajectories that start in the G1 phase and end in the G0 phase. Also, the weights associated with ‘incorrect’ and ‘did-not-start’ penalties were chosen to be 2^*N*^/*N*_*G*1_ so that these penalties scales with the number of nodes in the network. Note, based on the definition of the network score, in networks with relatively smaller score, the model dynamics follows the correct cell cycle trajectory with high frequency.

## Supporting information

S1 Table Statistical data related to perturbation analysis of cell cycle model in budding yeast cells. This Excel file contains statistical data related to the single-edge (sheet 1) and double-edge (sheet 2) perturbation analysis of Model A (cell cycle in budding yeast based on Ref. [11]). The data was calculated after performing 500 iterations per single-edge perturbations and 20 iterations per double perturbation. Sheet 3 shows the final states (attractors) of the model after and their corresponding sizes.
(XLSX)

S2 Table Statistical data related to perturbation analysis of cell cycle model in mammalian cells. This Excel file contains statistical data related to the single-edge (sheet 1) and double-edge (sheet 2) perturbation analysis of Model B (cell cycle in mammalian cell based on the Tyson-Novak model [1]). The data was calculated after performing 500 iterations per single-edge perturbations and 20 iterations per double perturbation. Sheet 3 shows the final states (attractors) of the model after and their corresponding sizes.
(XLSX)

S3 Table Statistical data related to perturbation analysis of cell cycle model in mammalian cells. This Excel file contains statistical data related to the single-edge (sheet 1) and double-edge (sheet 2) perturbation analysis of Model C (cell cycle in mammalian cell based on the Goldbeter model [2]). The data was calculated after performing 300 iterations per single-edge perturbations and 8 iterations per double perturbation. Sheet 3 shows the final states (attractors) of the model after and their corresponding sizes.
(XLSX)

S1 Text Description of details of simulation methods and statistical analysis. (DOCX)
