# Supplementary material for: A perturbation approach for refining Boolean models of cell cycle regulation
Source: PLoS One. 2024 Sep 6;19(9):e0306523. doi: 10.1371/journal.pone.0306523 (PMC11379194; doi:10.1371/journal.pone.0306523)
Supplement: S1 Text — (DOCX) [file pone.0306523.s004.docx]

## **Supporting Information for: A Perturbation Approach for Refining Boolean Models of Cell Cycle Regulation**

**S1 Table**

This Excel file contains statistical data related to the single-edge (sheet 1) and double-edge (sheet 2) perturbation analysis of Model A (cell cycle in budding yeast based on Ref. (1)). The data was calculated after performing 500 iterations per single-edge perturbations and 20 iterations per double perturbation. Sheet 3 shows the final states (attractors) of the model after and their corresponding sizes. The calculation of steady states is described in the execution flow below.

**S2 Table**

This Excel file contains statistical data related to the single-edge (sheet 1) and double-edge (sheet 2) perturbation analysis of Model B (cell cycle in mammalian cell based on the Tyson-Novak model (2)). The data was calculated after performing 500 iterations per single-edge perturbations and 20 iterations per double perturbation. Sheet 3 shows the final states (attractors) of the model after and their corresponding sizes. The calculation of final states is described in the execution flow below.

**S3 Table**

This Excel file contains statistical data related to the single-edge (sheet 1) and double-edge (sheet 2) perturbation analysis of Model C (cell cycle in mammalian cell based on the Goldbeter model (3)). The data was calculated after performing 300 iterations per single-edge perturbations and 8 iterations per double perturbation. Sheet 3 shows the final states (attractors) of the model after and their corresponding sizes. The calculation of final states is described in the execution flow below.

**Description of columns in the Supporting Excel Files**

**Perturbation:** This column lists the perturbations to the original model, and the original model is denoted by Original Model in this column. For other rows, the column follows the pattern *<source_node>-to-<target_node> -> <from_value>to<to_value>*, and for double perturbations the patterns are joined by the symbol | to signify separate perturbation. For example, CycA-to-CycE → 0 to1 | CycA-to-P21 → 0 to -1 means that for this particular perturbation we changed the weight of the edge going from Cyclin A (CycA) to Cyclin E (CycE) from 0 to 1 AND the weight of the edge going from Cyclin A (CycA) to P21-P27 (P21) from 0 to -1.

**Database Check:** This column can only be found for mammalian models (Model B and Model C). We could not find similar data for yeast model. We checked the validity of the perturbations generated by simulations by comparing the interaction against the protein-protein interaction database SIGNOR (https://signor.uniroma2.it). For the case of a single perturbation, we checked if the corresponding entity pair (node1, node2, interaction) exists in the database. If they do, we mark the perturbation as TRUE, and otherwise FALSE. In cases of self-loops (edge going from a node to itself), we did not find any information in the database. In such cases we mark the column as NA. Also, if the perturbation is the removal of an edge (perturbation from 1/ -1 to 0), and we do not find any interaction between those two nodes in the database, then we mark the column as TRUE as it implies there is no interaction between those two nodes. In case of double perturbation, we mark this column as TRUE only if both the perturbations align with the results from the database. If one of the perturbations in double perturbation is self-loop, we ignore the self-loop and mark this column based on the result from the other perturbation. Following the same example from the previous section: “CycA-to-CycE → 0 to 1”, we observe that the weight going from Cyclin A (CycA) to Cyc E (CycE) was changed from 0 to 1. The corresponding entity in the database would be any ONE of "CyclinA2/CDK2", 'CDK2', 'CCNA1' in the database and similarly the corresponding entity for Cyclin E (CycE) would be any one of "CyclinB/CDK1", "CDK1", 'CCNB1' in the database. We check all 6 possible matches for a signal of upregulation (see section Database Validation Script) since the interaction is now classified as 1. If we find such a match, we mark this perturbation as True, if not, we mark it as False. For the case of 0, we similarly check all database matches and ensure there is no possible match in the database before marking the entry as True.

**Reference:** This column we provide some context for the perturbations for which we find a match in the database. The format of the 'Reference' column is '*[<db_source_node> -> <db_target_node> -> <interaction_type> -> <pubmed_id(s)>]*'. Here db_source_node is the node name found in the database that matches with the source node in the perturbation, db_target_node is the node name found in the database that matches with the target node in the perturbation, interaction_type can be either 1 or -1 based on their interaction type (activation/repression). The pubmed_id(s) are the PubMed identifier of the articles that support the interaction given in the database. In case of double perturbation, wherever possible, we write two such references in the same row.

**Graph Score:** We calculate the Graph Score based on the formula given in Perturbations and Scoring section under Methods.

**Normalized Graph Score:** To calculate the Normalized Graph Score, we divide the Graph Score of each perturbation with the score of the Original Model. For example, if we have the Graph Score for some perturbation as 1200, and the Graph Score for the Original Model of the same model is 800, then the Normalized Graph Score for the particular perturbation is (1200/ 800) = 1.5. By definition the Normalized Graph Score for the Original Model for each model is always 1.

**Steady State Count:** This is the average number of final state(s) for each model after executing them for a particular number of iterations. Because of the stochastic update method, and finite number of iterations, in some simulation runs the dynamics does not reach the steady state. Therefore, the steady state count can be slightly larger than the number of actual steady states of the model.

**Largest Basin Size:** This is the average size of the largest final state. For example, a graph with 10 nodes has $2^{10}=1024$ initial states, each of which reach some final state at the end of the simulation run. If there are 3 Steady States for this cycle with the distribution being 1000, 18, 6, then we say that the Largest Basin Size for this model to be 1000.

**Correct(%), Incorrect(%), Did not Start(%)**: These columns denote the percentage of trajectories (with initial condition limited to G1 states) that go via the correct cell cycle sequence, incorrect cell cycle sequence, or do-not-start the cell cycle at all, respectively. More details of the process to distinguish them are discussed in detail in the Sequence section under Methods header.

An example of Correct path for Model C:

| T | CycD | CycE | CycA | CycB | E2F | Skp2 | Cdh1 | Cdc25 | RB | P21 | Cdc20 | Wee1 | PPs |
| --- | --- | --- | --- | --- | --- | --- | --- | --- | --- | --- | --- | --- | --- |
| 1 | 1 | 0 | 0 | 0 | 0 | 1 | 0 | 0 | 1 | 1 | 0 | 1 | 0 |
| 2 | 1 | 1 | 0 | 0 | 0 | 1 | 0 | 0 | 1 | 1 | 0 | 1 | 0 |
| 3 | 0 | 0 | 0 | 0 | 0 | 1 | 0 | 0 | 1 | 1 | 0 | 1 | 0 |
| 4 | 0 | 0 | 0 | 0 | 1 | 1 | 0 | 0 | 1 | 1 | 0 | 1 | 0 |
| 5 | 0 | 1 | 0 | 0 | 1 | 1 | 0 | 0 | 1 | 1 | 0 | 1 | 0 |
| 6 | 0 | 0 | 0 | 0 | 1 | 1 | 0 | 0 | 1 | 1 | 0 | 1 | 0 |
| 7 | 0 | 0 | 1 | 0 | 1 | 1 | 0 | 0 | 1 | 1 | 0 | 1 | 0 |
| 8 | 0 | 0 | 1 | 1 | 1 | 1 | 0 | 0 | 1 | 1 | 0 | 1 | 0 |
| 9 | 0 | 0 | 1 | 0 | 1 | 1 | 0 | 0 | 1 | 1 | 0 | 1 | 0 |
| 10 | 0 | 0 | 0 | 0 | 1 | 1 | 0 | 0 | 1 | 1 | 0 | 1 | 0 |
| 11 | 0 | 0 | 0 | 0 | 1 | 1 | 0 | 0 | 1 | 1 | 1 | 1 | 0 |
| 12 | 0 | 0 | 0 | 0 | 1 | 1 | 0 | 0 | 1 | 1 | 0 | 1 | 0 |
| 13 | 0 | 0 | 0 | 0 | 1 | 1 | 1 | 0 | 1 | 1 | 0 | 1 | 0 |
| 14 | 0 | 0 | 0 | 0 | 1 | 0 | 1 | 0 | 1 | 1 | 0 | 1 | 0 |
| 15 | 0 | 0 | 0 | 0 | 0 | 0 | 1 | 0 | 1 | 1 | 0 | 1 | 0 |

Here we can see that CycE turns 1 at T=2, signaling that cell cycle has reached point-of-no-return. After that, it goes through the states in the expected order:

{E2F: 1, CycE: 0, CycA: 0, CycB: 0, Cdc20: 0} (at T=4),

{CycE: 1, CycA: 0, CycB: 0, Cdc20: 0} (at T=5),

{CycA: 1, CycB: 0, Cdc20: 0} (at T=7),

{CycB: 1, Cdc20: 0} (at T=8),

{Cdc20: 1} (at T=11).

An example of Incorrect path for Model C:

| T | CycD | CycE | CycA | CycB | E2F | Skp2 | Cdh1 | Cdc25 | RB | P21 | Cdc20 | Wee1 | PPs |
| --- | --- | --- | --- | --- | --- | --- | --- | --- | --- | --- | --- | --- | --- |
| 1 | 1 | 0 | 0 | 0 | 0 | 1 | 1 | 0 | 1 | 1 | 0 | 1 | 0 |
| 2 | 1 | 0 | 0 | 0 | 0 | 1 | 0 | 0 | 1 | 1 | 0 | 1 | 0 |
| 3 | 1 | 1 | 0 | 0 | 0 | 1 | 0 | 0 | 1 | 1 | 0 | 1 | 0 |
| 4 | 1 | 0 | 0 | 0 | 1 | 1 | 0 | 0 | 1 | 1 | 0 | 1 | 0 |
| 5 | 1 | 1 | 0 | 0 | 1 | 1 | 0 | 0 | 1 | 1 | 0 | 1 | 0 |
| 6 | 1 | 0 | 0 | 0 | 1 | 1 | 0 | 0 | 1 | 1 | 0 | 1 | 0 |
| 7 | 1 | 0 | 0 | 0 | 1 | 1 | 0 | 0 | 1 | 1 | 0 | 1 | 0 |
| 8 | 0 | 0 | 0 | 1 | 1 | 1 | 0 | 0 | 1 | 1 | 0 | 1 | 0 |
| 9 | 0 | 0 | 0 | 0 | 1 | 1 | 0 | 0 | 1 | 1 | 0 | 1 | 0 |
| 10 | 0 | 0 | 0 | 0 | 1 | 1 | 0 | 0 | 1 | 1 | 0 | 1 | 0 |
| 11 | 0 | 0 | 0 | 0 | 1 | 1 | 0 | 0 | 1 | 1 | 1 | 1 | 0 |

Here we can see that CycE turns 1 at T=3, but after that it does not follow the correct sequence in the expected order before reaching the end. Even though it reaches first and second expected states at T=4 and T=5, it does not proceed to other expected states after that.

An example of Did not Start path for Model C:

| T | CycD | CycE | CycA | CycB | E2F | Skp2 | Cdh1 | Cdc25 | RB | P21 | Cdc20 | Wee1 | PPs |
| --- | --- | --- | --- | --- | --- | --- | --- | --- | --- | --- | --- | --- | --- |
| 1 | 1 | 0 | 0 | 0 | 0 | 1 | 1 | 0 | 1 | 1 | 0 | 1 | 0 |
| 2 | 0 | 0 | 0 | 0 | 0 | 1 | 1 | 0 | 1 | 1 | 0 | 1 | 0 |
| 3 | 0 | 0 | 0 | 0 | 0 | 1 | 0 | 0 | 1 | 1 | 0 | 1 | 0 |
| 4 | 0 | 0 | 0 | 0 | 1 | 1 | 0 | 0 | 1 | 1 | 0 | 1 | 0 |
| 5 | 0 | 0 | 0 | 0 | 1 | 1 | 0 | 0 | 1 | 0 | 0 | 1 | 0 |
| 6 | 0 | 0 | 0 | 0 | 1 | 1 | 1 | 0 | 1 | 0 | 0 | 1 | 0 |
| 7 | 0 | 0 | 0 | 0 | 1 | 0 | 1 | 0 | 1 | 0 | 0 | 1 | 0 |
| 8 | 0 | 0 | 0 | 0 | 0 | 0 | 1 | 0 | 1 | 0 | 0 | 1 | 0 |

Here even though we see that the cell cycle quickly reached the expected final state corresponding to the state: {CycD: 0, CycE: 0, CycA: 0, CycB: 0, E2F: 0, Skp2: 0, Cdh1: 1, Cdc25: 0, RB: 1, P21: 0/1, Cdc20: 0, Wee1: 1, PPs: 0}, CycE never got to 1 state. Hence the cell cycle starting signal was not generated, and we consider this case to be a Did-not-Start case.

Note: As the original state tables for each state can become quite large, we have skipped a few intermediate states in the example tables above. The tables above are only to illustrate how the sequence checking works in the algorithm.

**Most Frequent Steady State(s):** This is the state corresponding to the largest attractor for each graph.

# Execution Flow of the cell cycle:

Our method to execute the simulations for each model and gather statistical data is shown in the scheme below. The For each model with $N$ nodes, there can be a maximum of $2^{N}$ initial conditions (IC1, IC2, … , IC$2^{N}$), which are simulated to generate a score for that execution using the formula given in the Perturbation and Scoring section. We repeat the steps multiple times, gathering scores and other relevant data for every complete execution. The number of execution (x) to gather this statistical data varies based on the time taken to execute a complete cycle. After gathering data from all such x executions, we perform statistical operations on the data to make them presentable.


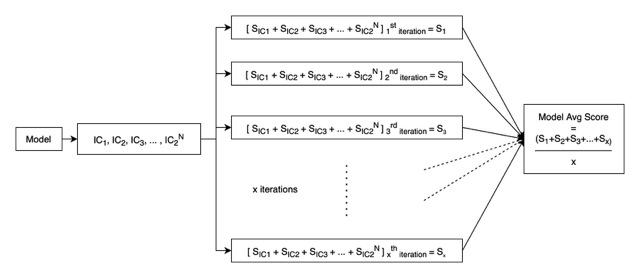


Execution Flow for a single model

Due to computational limitations, we do not execute every cell cycle till it reaches a true steady state. Rather we impose an upper bound on the number of updates each cell cycle can have under different conditions. For example, when we are calculating the performance of all perturbations we limit each cell cycle to a maximum of $(100* Number of nodes)$ updates. That means, for each initial state $(IC1, IC2 ... IC2^{N})$, we make updates to the cell cycle for $(100* Number of nodes)$ times and the final state achieved after these updates are considered the steady states for the initial states. Again, when we are looking for the true steady states, we have to let the cell cycle run for a lot more steps, sometimes around $(500\times Number of nodes)$ times. Even after all these updates, we have to manually take another look at the final states and individually analyze them to verify if they are true steady states or not.

**Steady States:** As the update method is stochastic in nature, due to finite number of iterations, the number of steady states can differ between two simulation runs. We put an upper limit on the number of iterations to narrow down the final steady states, and then checked for the true steady states by choosing this set as the initial condition and increasing the number of iterations.

**Cycle Detection:** The model can also detect whether there is a simple cycle at the end of a cell cycle. For any initial state, we run the cell cycle for at least 100*n times, where n is the number of nodes in each model. After the cell cycle is iterated for the number of given iterations, if sequences of states repeats in the same order multiple time, we conclude that the cell cycle has gone into a cycle and define the final state as “C” for every node, e.g. (CycD: C, CycE: C, … ). Even though we tried detecting most of the cycles, there are more convoluted cycles visible in the first mammal model that are very difficult to identify.

**Database Validation Script**

This is a script written in the Python programming language. It checks the perturbations generated by simulations against the database - SIGNOR 3.0 [https://signor.uniroma2.it/], and adds the 2 columns – Reference and Database Check. This script is executed only for Model B and C as the database does not contain data for Model A.

From the SINGNOR 3.0 Database, we extract the ENTITY A and ENTITY B fields, corresponding to the source node and target node, respectively, and the interaction between them. The interactions are grouped into upregulation and downregulation, where these expressions include the following classes from the database:

upregulation: (up-regulates, up-regulates activity, up-regulates quantity, up-regulates quantity by expression, up-regulates quantity by stabilization)

downregulation: (down-regulates, down-regulates activity, down-regulates quantity, down-regulates quantity by repression, down-regulates quantity by destabilization)

Using this logic, the script first extracts the set of known perturbations from the database. It then checks the perturbations that were generated in our simulations against this known map.

Single Perturbation Evaluation-

1. The script checks if the perturbation of the type (A, B, up) or (A, B, down), suggested by the simulation, exists in the database. If it does, we classify the perturbation as TRUE, and otherwise FALSE.
2. For the perturbation where an interaction goes to 0 (e.g. A to B: 1 → 0), we mark this perturbation as TRUE if it does not exist in the database.
3. If we find a matching entry in the database, we add the PMID from the database to the Reference column. This provides information to identify the exact item that matched in the database.
4. Self-loops (same target and source node) are considered invalid for this check. A row result (Reference and Database Check) for perturbations containing self-loops should be ignored.

Double Perturbation Evaluation -

1. Each perturbation is evaluated with the same rules as for a single perturbation.
2. If both perturbations are in the database, then it is classified as TRUE.
3. If there is a self-loop, that perturbation is ignored, and the other (non-self-loop) perturbation is counted towards the result. The result of non-self-loop perturbations is aggregated with logical AND - only marked true if all non-self-loop perturbations exist in the database.

**Statistics of Perturbations**

Model A

| Score Range | Single Perturbation | Double Perturbation |
| --- | --- | --- |
| Less than 1 (<1) | 28.01% | 14.94% |
| Greater than or Equal to 1 (>=1) | 72.19% | 85.06% |

Model B

| Score Range | DB Check | Single Perturbation | | Double Perturbation | |
| --- | --- | --- | --- | --- | --- |
| Less than 1 (<1) | True | 39.5% | 5.06% | 35.2% | 2.34% |
|  | NA |  | 12.66% |  | 0.99% |
|  | False |  | 82.28% |  | 96.67% |
| Greater than or equal to 1 (>=1) | True | 60.5% | 14.05% | 64.8% | 3.63% |
|  | NA |  | 8.26% |  | 0.87% |
|  | False |  | 77.69 |  | 95.5 |

Model C

| Score Range | DB Check | Single Perturbation | | Double Perturbation | |
| --- | --- | --- | --- | --- | --- |
| Less than 1 (<1) | True | 35.2% | 14.3% | 2% | 2.72% |
|  | NA |  | 2.5% |  | 0.09% |
|  | False |  | 83.2% |  | 97.19% |
| Greater than or equal to 1 (>=1) | True | 64.8% | 12.33% | 98% | 3.69% |
|  | NA |  | 10.5% |  | 0.56% |
|  | False |  | 77.17% |  | 95.75% |

**References**

1. Li F, Long T, Lu Y, Ouyang Q, Tang C. The yeast cell-cycle network is robustly designed. Proc Natl Acad Sci U S A. 2004;101(14):4781-6.

2. Novak B, Tyson JJ. A model for restriction point control of the mammalian cell cycle. J Theor Biol. 2004;230(4):563-79.

3. Gerard C, Goldbeter A. Temporal self-organization of the cyclin/Cdk network driving the mammalian cell cycle. Proc Natl Acad Sci U S A. 2009;106(51):21643-8.
