# Supplementary material for: A perturbation approach for refining Boolean models of cell cycle regulation
Source: PLoS One. 2024 Sep 6;19(9):e0306523. doi: 10.1371/journal.pone.0306523 (PMC11379194; doi:10.1371/journal.pone.0306523)
Supplement: Attachment — Submitted filename: Response to Reviewers.docx [file pone.0306523.s005.docx]

Dear Editor,

Thank you for giving us the opportunity to revise our manuscript. We are thankful to the reviewers for their constructive comments and suggestions. By addressing them the manuscript has improved significantly. Our responses to all the questions raised by the reviewers are given below:

**Response to Reviewer #1:** 
 
1. Figures representing the original networks are unreadable

We have improved the resolution of all Figures. The submitted figures meet the PLOS ONE quality guidelines.

2. Several elements lack clear definitions and explanations. For instance, the mention of "very complicated" trajectories for the asynchronous update schemes (lines 53-56) lacks elaboration (it is not clear what does it mean to be “very complicated”),

We agree with the reviewer. We have removed the term “very complicated” and stated that a limitation of asynchronous updated scheme is that the run time of simulations increases rapidly with the number of nodes in the network.

3. The score function's definition is unclear due to the ambiguous concept of the "k-th component" for a steady state. In the context of graph theory, a connected component of an undirected graph (or a strongly connected component of a directed graph) does not correspond to a specific numerical value but to a maximal connected subgraph (maximal strongly connected subgraph in the case of a strongly connected component). I assume that in the context of the paper it may refer to the convergence time starting from a particular initial condition to a specific fixed point but, it is not clear from the text

We have added sentences to make the concept of "k-th component" clearer (see lines 317-320).

The state of the Boolean network at any given time is described by an N-component vector, where the components are the activities of different proteins in the network. The k-th component refers to the k-th element of that vector. For example, in the budding yeast model (Model A) the 1^st^ component is the activity of Cln3 and the 5^th^ component is the activity of Cdh1.

4. Additionally, the idea behind the definition of the score as a measure of model reliability is not well-explained, beyond the concept of penalizing "incorrect" and "did-not-start" trajectories.

We have improved the paragraph in which the network score is defined (see lines 375-379). Briefly, the main aim of our work is to develop Boolean networks of cell cycle in which the dynamics follows the correct cell cycle trajectory with high frequency. The network score defined in the Methods section captures this aspect of the dynamics. Meaning, for networks with lower score (than the original network) the dynamics follows the correct cell cycle trajectory with higher frequency. Hence, we think, the network score is a good representation of the reliability of the model (where reliability is defined as the frequency with which the dynamics follows the correct cell cycle trajectory)

5. The automatic aspect of the method requires more emphasis and comparison with other approaches. For example, it could be interesting to explore a potential connection with the concept of the neutral space used in [1].

We have added a paragraph in the Discussion section (see lines 293-302) describing the automated aspects of the model in more detail. Briefly, by combining our perturbation approach with an automated database search, we can refine Boolean models of cell cycle. We also suggest a simple method for adding new genes to the existing network. This process of model refinement and growth (adding new genes), in principle, does not require any intervention from the user, and therefore can serve as a standardized method for building data-centric Boolean models of cell cycle. In reality, however, the information available in databases are incomplete. Hence, in our work manual checks on the validity of interaction were performed.

We read through the reference on neutral space. Although the idea is very interesting, we couldn’t find its direct connection to our work.

6. Typos: 333 - it says $2N^2$ and I think it should be $2^{N^{2}}.$

This is not a typo. The total number of single-edge perturbations (including perturbation to self-loops) is indeed 2N^2^, for a network with N nodes.

Number of ways an edge can be selected for perturbation = N*N = N^2^

Number of ways the selected edge can be perturbed = 2. As there are only 3 possibilities for the value of any edge (-1, 0, 1), any perturbation to any edge can have 2 possibilities.

So, the total number of single-edge perturbations = 2*N^2^.

**Response to Reviewer #2:** 
 
1. Further information on how the proposed method compares to existing methods of model refinement would be valuable.

To the best of our knowledge there are no model refinement methods related to Boolean models of cell cycle. All existing models focus on the dynamics of a fixed network. In Li et. al., the perturbation analysis was used, but as a means to study the stability of the network, and not as a means for refining it.

2. A discussion on the potential advantages and limitations of the perturbation approach would provide greater context for the significance of the findings. 

We have added a paragraph in the Discussion section (see lines 284-291), describing the advantages and limitations of our approach. Briefly, the main advantage of our method is that it is a step forward towards building data-centric Boolean models of cell cycle, as opposed to user-centric models where the user decides the nodes and interactions to include in the model. Our data-centric approach relies on comparing the interaction corresponding to a perturbation with those available in the protein-protein interaction database SIGNOR.

A limitation of our approach is that it relies on the data available on SIGNOR database, which we found to be incomplete (examples are provided).

3. The authors should consider providing a clearer explanation of how the proposed interactions are validated against experimental data. The use of the SIGNOR 3.0 database is mentioned, but a more detailed description of this validation process may be beneficial for the readers (I acknowledge that my understanding on the database may not be comprehensive). 

A more detailed description of the validation process, along with one specific example, is provided in the Supporting Information document. See the section Database Check in Supplementary Information.

4. Details of how the method could be fully automated in the future would be a valuable addition. This would offer insight into the potential scalability and applicability of the approach for other networks and systems. 

We have added paragraphs in the Discussion section describing how the method could be fully automated (see lines 289-302). Briefly, the model development process involves two aspects: model growth and model refinement. We have described methods by which both processes can be carried out fully automatically. The methods rely on database information being fully correct and up-to-date, which currently is not the case. But, as their accuracy increases, our proposed method will become fully automated.

5. It would be beneficial to enhance the visual clarity and resolution of the graphs for better comprehension. 

We have improved the resolution of all Figures. The submitted figures meet the PLOS ONE quality guidelines

6. Finally, the manuscript would be strengthened by a critical discussion of the limitations and potential future directions of the research.

We have added a paragraph in the Discussion section (lines 293-302), describing the future direction of our research. Briefly, our plan is to introduce additional cell cycle gene into the existing network. We have described a simple method to achieve this objective. We have also described the limitations of the model (lines 284-291). The main limitation of our approach is its reliance on the information available in the SIGNOR database, which, currently, is not up-to-date and sometimes incorrect. But, with improving quality of the databases, we think that issue will go away.
