# Supplementary material for: A perturbation approach for refining Boolean models of cell cycle regulation
Source: PLoS One. 2024 Sep 6;19(9):e0306523. doi: 10.1371/journal.pone.0306523 (PMC11379194; doi:10.1371/journal.pone.0306523)
Supplement: Attachment — Submitted filename: Response to Reviewers.docx [file pone.0306523.s006.docx]

Dear Editor,

Thank you for giving us the opportunity to revise our manuscript. We thank the reviewers for reading our revised manuscript and for their constructive comments and suggestions. Our responses to all the questions raised by the reviewers are given below:

**Response to Reviewer #1**

1. Although the resolution of the images has improved considerably, Figure 1 is still difficult to understand. I think the authors should rework this figure to make it clearer.

We have improved the resolution of Figure 1 even more. Now the resolution of the figure is 400 dpi, well above the minimum requirement of 300 dpi. We request the reviewer to see the stand-alone figure file figure1.tiff in our submission. The figure can look grainy when inserted into a pdf file.

2. Although it was added in the introduction that: "A drawback of the asynchronous scheme is that the run time for simulations increases rapidly with the number of nodes in the network," no references are provided to support it. It would be interesting to add references from other papers that theoretically or experimentally justify the statement.

Thank you for the suggestion. We have added a reference (reference 16, Garg et. al., Bioinformatics, 2008) to support the statement. Data in Table 1 of this reference shows that the run time of simulation increases with the number of nodes and edges in the network.

3. In addition, regarding the following statement: “Asynchronous update preserved the G0 steady state, but the cyclic attractor became very complex with many intertwined cycles.” The authors should provide more details in what does it mean “cyclic attractor” in the context of stochastic dynamics.

This statement was put to describe a result from the article by Faure et. al. (reference 13), in which the authors investigated a Boolean model of mammalian cell cycle. They found that in the context of stochastic dynamics, the attractor is not cyclic. It is instead a complex attractor made up of the cyclic attractor observed in the case of synchronous (deterministic) update scheme, plus many other states. We have edited the sentence to make it clear that in the context of stochastic dynamics the attractor is not cyclic. Here is a more detailed description of the result from Faure et. al.:

Using synchronous update method, the authors found that upon activation of Cyclin D (needed to start the cell cycle), the dynamics went through a cycle made off 7 successive states (Figure 2, bottom left panel in the reference). When the update method was changed to asynchronous, the cyclic attractor with 7 states changed to an attractor with 112 states (Figure 2, top panel in the reference). This large attractor contained 7 states forming the synchronous cycle plus many components involving smaller number of states (representing transient oscillations according to the authors).

4. I still think that the paper could be greatly improved if it is better situated in the context of the state of the art. How does it compare with other methods?

We have added sentences in the Discussion section (see lines 276-280) to explain how our work fits into the context of the state of art.

Briefly, our work uses of single-edge and double-edge perturbations to refine the network of interaction describing the cell cycle. Edge perturbations have been used in several other works including the study different classes of mutations in human inherited disorders (Zhong et. al., 2009), genotype-phenotype associations using alleles that lack single interactions in C. elegans (Dreze et. al., 2009), removal of unwanted attractors such as the cancerous attractor in T-LGL leukemia (Campbell et. al., 2019), and the stability of the largest attractor in budding yeast cell cycle (Li et. al., 2004). The study by Campbell et. al., involving removal of unwanted attractors comes closest to ours in terms of methodology. Our method however is designed not only to remove the unwanted attractors, but also to make sure that the cell cycle dynamics follows the correct path as it reaches the desired correct attractor. Furthermore, unlike other approaches, our aim was to develop a method that enables the automation of the model refinement process.
