# Supplementary material for: A perturbation approach for refining Boolean models of cell cycle regulation
Source: PLoS One. 2024 Sep 6;19(9):e0306523. doi: 10.1371/journal.pone.0306523 (PMC11379194; doi:10.1371/journal.pone.0306523)
Supplement: Peer review history

# Peer review: Decision Letter 0

**DOI:** 10.1371/journal.pone.0306523.r001

**Authors:** Gonzalo A. Ruz

25 Jan 2024

PONE-D-23-41629A Perturbation Approach for Refining Boolean Models of Cell Cycle RegulationPLOS ONE

Dear Dr. Banerjee,

Thank you for submitting your manuscript to PLOS ONE. After careful consideration, we feel that it has merit but does not fully meet PLOS ONE’s publication criteria as it currently stands. Therefore, we invite you to submit a revised version of the manuscript that addresses the points raised during the review process.

Please submit your revised manuscript by Mar 10 2024 11:59PM. If you will need more time than this to complete your revisions, please reply to this message or contact the journal office at plosone@plos.org. When you're ready to submit your revision, log on to https://www.editorialmanager.com/pone/ and select the 'Submissions Needing Revision' folder to locate your manuscript file.

Please include the following items when submitting your revised manuscript:

- A rebuttal letter that responds to each point raised by the academic editor and reviewer(s). You should upload this letter as a separate file labeled 'Response to Reviewers'.
- A marked-up copy of your manuscript that highlights changes made to the original version. You should upload this as a separate file labeled 'Revised Manuscript with Track Changes'.
- An unmarked version of your revised paper without tracked changes. You should upload this as a separate file labeled 'Manuscript'.

If you would like to make changes to your financial disclosure, please include your updated statement in your cover letter. Guidelines for resubmitting your figure files are available below the reviewer comments at the end of this letter.

If applicable, we recommend that you deposit your laboratory protocols in protocols.io to enhance the reproducibility of your results. Protocols.io assigns your protocol its own identifier (DOI) so that it can be cited independently in the future. For instructions see: https://journals.plos.org/plosone/s/submission-guidelines#loc-laboratory-protocols. Additionally, PLOS ONE offers an option for publishing peer-reviewed Lab Protocol articles, which describe protocols hosted on protocols.io. Read more information on sharing protocols at https://plos.org/protocols?utm_medium=editorial-email&utm_source=authorletters&utm_campaign=protocols.

We look forward to receiving your revised manuscript.

Kind regards,

Gonzalo A. Ruz, Ph.D.

Academic Editor

PLOS ONE

Journal requirements:

When submitting your revision, we need you to address these additional requirements.

1. Please ensure that your manuscript meets PLOS ONE's style requirements, including those for file naming. The PLOS ONE style templates can be found at

https://journals.plos.org/plosone/s/file?id=wjVg/PLOSOne_formatting_sample_main_body.pdf and

https://journals.plos.org/plosone/s/file?id=ba62/PLOSOne_formatting_sample_title_authors_affiliations.pdf

2. Please note that PLOS ONE has specific guidelines on code sharing for submissions in which author-generated code underpins the findings in the manuscript. In these cases, all author-generated code must be made available without restrictions upon publication of the work. Please review our guidelines at https://journals.plos.org/plosone/s/materials-and-software-sharing#loc-sharing-code and ensure that your code is shared in a way that follows best practice and facilitates reproducibility and reuse.

3. Thank you for stating the following in the Acknowledgments Section of your manuscript:

“The work was supported by the Academy of Data Science Discovery Fund awarded to AB and PK.”

We note that you have provided funding information that is not currently declared in your Funding Statement. However, funding information should not appear in the Acknowledgments section or other areas of your manuscript. We will only publish funding information present in the Funding Statement section of the online submission form.

Please remove any funding-related text from the manuscript and let us know how you would like to update your Funding Statement. Currently, your Funding Statement reads as follows:

“The author(s) received no specific funding for this work.”

Please include your amended statements within your cover letter; we will change the online submission form on your behalf.

4. Please include captions for your Supporting Information files at the end of your manuscript, and update any in-text citations to match accordingly. Please see our Supporting Information guidelines for more information: http://journals.plos.org/plosone/s/supporting-information.

Additional Editor Comments:

Both reviewers have raised important issues, which the authors must address.

[Note: HTML markup is below. Please do not edit.]

Reviewers' comments:

Reviewer's Responses to Questions

**Comments to the Author**

1. Is the manuscript technically sound, and do the data support the conclusions?

The manuscript must describe a technically sound piece of scientific research with data that supports the conclusions. Experiments must have been conducted rigorously, with appropriate controls, replication, and sample sizes. The conclusions must be drawn appropriately based on the data presented. 

Reviewer #1: No

Reviewer #2: Yes

**********

2. Has the statistical analysis been performed appropriately and rigorously? 

Reviewer #1: N/A

Reviewer #2: Yes

**********

3. Have the authors made all data underlying the findings in their manuscript fully available?

The PLOS Data policy requires authors to make all data underlying the findings described in their manuscript fully available without restriction, with rare exception (please refer to the Data Availability Statement in the manuscript PDF file). The data should be provided as part of the manuscript or its supporting information, or deposited to a public repository. For example, in addition to summary statistics, the data points behind means, medians and variance measures should be available. If there are restrictions on publicly sharing data—e.g. participant privacy or use of data from a third party—those must be specified.

Reviewer #1: Yes

Reviewer #2: Yes

**********

4. Is the manuscript presented in an intelligible fashion and written in standard English?

PLOS ONE does not copyedit accepted manuscripts, so the language in submitted articles must be clear, correct, and unambiguous. Any typographical or grammatical errors should be corrected at revision, so please note any specific errors here.

Reviewer #1: Yes

Reviewer #2: Yes

**********

5. Review Comments to the Author

Please use the space provided to explain your answers to the questions above. You may also include additional comments for the author, including concerns about dual publication, research ethics, or publication ethics. (Please upload your review as an attachment if it exceeds 20,000 characters)

Reviewer #1: TITLE: A Perturbation Approach for Refining Boolean Models of Cell Cycle Regulation

AUTHORS: Anand Banerjee, Asif Iqbal Rahaman, Alok Mehandale, Pavel Kraikivski

----------- Recommendation -----------

Major revision

----------- Overall Evaluation -------------------------

Summary: The paper focuses on the challenge of constructing models for large protein regulatory networks, with a particular emphasis on boolean network models. This model is composed by a directed signed graph, where each protein is represented by a node. Nodes can be active (state 1) or inactive (state 0), with regulatory dependencies depicted by directed edges. A $-1$ label on an edge indicates an inhibition link, while +1 denotes an activation link. Local functions in the paper are threshold functions i.e. the future state of a node depends on the sum of the states of its in-neighbors multiplied by the sign of its corresponding edges. Each node (representing a protein) needs this sum to surpass certain threshold in order to activate. In the paper, activation occurs when the sum of the states of the in-neighbors of a node is positive; non-activation results from a negative sum. If the sum is 0, the state of the node remains unchanged. Dynamics are defined asynchronously by randomly selecting a node for update in each time step. The paper proposes a "semi-automatic approach" for constructing reliable models, using single-edge and double-edge perturbations, involving changes in edge signs. These perturbation protocols are simply defined by a change on the sign of an edge (or two edges for the double edge perturbation). More precisely, the single edge perturbation protocol consists on one of the following operations: i) to change the sign of an edge from -1 to 1 (or symmetrically from 1 to -1) or ii) to remove an edge or adding an edge (wether a positive or a negative edge). Double-edge perturbations are simply single perturbations done on two edges at the same time.

The proposed method can be summarized in four main steps:

(a) Manually construct an initial Boolean model using existing Boolean or ODE models of the cell cycle.

(b) Define a network score to quantify the consistency of network dynamics with the cell cycle.

(c) Use single-edge and double-edge perturbations to find interactions resulting in a lower score.

(d) Compare proposed interactions with databases to identify those consistent with experimental data.

While the main idea is interesting, the paper's presentation is inadequate. Figures representing the original networks are unreadable, and several elements lack clear definitions and explanations. For instance, the mention of "very complicated" trajectories for the asynchronous update schemes (lines 53-56) lacks elaboration (it is not clear what does it mean to be “very complicated”), and the score function's definition is unclear due to the ambiguous concept of the "k-th component" for a steady state. In the context of graph theory, a connected component of an undirected graph (or a strongly connected component of a directed graph) does not correspond to a specific numerical value but to a maximal connected subgraph (maximal strongly connected subgraph in the case of a strongly connected component). I assume that in the context of the paper it may refer to the convergence time starting from a particular initial condition to a specific fixed point but, it is not clear from the text. Additionally, the idea behind the definition of the score as a measure of model reliability is not well-explained, beyond the concept of penalizing "incorrect" and "did-not-start" trajectories.

The automatic aspect of the method requires more emphasis and comparison with other approaches. For example, it could be interesting to explore a potential connection with the concept of the neutral space used in [1].

Considering these weaknesses, the paper is not suitable for publication in its current state. However, with improvements in presentation and addressing the latter issues, it could become a valuable contribution.

Score:

Positive Aspects:

• The main idea of the paper is original and interesting.

• The paper's organization is good.

Negative Aspects:

• Presentation is subpar; figures are poorly formatted and unreadable.

• Critical concepts like "k-th component" and "more complicated" trajectories lack clear definitions.

• The automatic aspect of the approach needs greater emphasis and comparison with other methods.

Typos: 333 - it says $2N^2$ and I think it should be $2^{N^{2}}.$

References

[1] Travisany, D., Goles, E., Latorre, M., Cortés, M. P., & Maass, A. (2020). Generation and robustness of Boolean networks to model Clostridium difficile infection. Natural Computing, 19, 111-134.

Reviewer #2: The manuscript presents an interesting study that applies a perturbation approach to refine Boolean models for cell cycle regulation. The approach is well-defined, and the methods used for building the models and analyzing the data seem robust and appropriate. The conclusions drawn from the study are supported by the data presented.

However, there are several areas where further clarification and additional information would strengthen the manuscript:

- Further information on how the proposed method compares to existing methods of model refinement would be valuable. A discussion on the potential advantages and limitations of the perturbation approach would provide greater context for the significance of the findings.

- The authors should consider providing a clearer explanation of how the proposed interactions are validated against experimental data. The use of the SIGNOR 3.0 database is mentioned, but a more detailed description of this validation process may be beneficial for the readers (I acknowledge that my understanding on the database may not be comprehensive).

- Details of how the method could be fully automated in the future would be a valuable addition. This would offer insight into the potential scalability and applicability of the approach for other networks and systems.

- It would be beneficial to enhance the visual clarity and resolution of the graphs for better comprehension.

Finally, the manuscript would be strengthened by a critical discussion of the limitations and potential future directions of the research.

**********

6. PLOS authors have the option to publish the peer review history of their article (what does this mean?). If published, this will include your full peer review and any attached files.

If you choose “no”, your identity will remain anonymous but your review may still be made public.

**Do you want your identity to be public for this peer review?** For information about this choice, including consent withdrawal, please see our Privacy Policy.

Reviewer #1: No

Reviewer #2: **Yes: **Luis Gómez Guzmán

**********

[NOTE: If reviewer comments were submitted as an attachment file, they will be attached to this email and accessible via the submission site. Please log into your account, locate the manuscript record, and check for the action link "View Attachments". If this link does not appear, there are no attachment files.]

While revising your submission, please upload your figure files to the Preflight Analysis and Conversion Engine (PACE) digital diagnostic tool, https://pacev2.apexcovantage.com/. PACE helps ensure that figures meet PLOS requirements. To use PACE, you must first register as a user. Registration is free. Then, login and navigate to the UPLOAD tab, where you will find detailed instructions on how to use the tool. If you encounter any issues or have any questions when using PACE, please email PLOS at figures@plos.org. Please note that Supporting Information files do not need this step.

# Author comment: Author response to Decision Letter 0

**DOI:** 10.1371/journal.pone.0306523.r002

29 Apr 2024

We have submitted a file labeled 'Response to Reviewers' in which we have addressed all the suggestions and questions raised by the reviewers.

Attachment Submitted filename: Response to Reviewers.docx

# Peer review: Decision Letter 1

**DOI:** 10.1371/journal.pone.0306523.r003

**Authors:** Gonzalo A. Ruz

27 May 2024

PONE-D-23-41629R1A Perturbation Approach for Refining Boolean Models of Cell Cycle RegulationPLOS ONE

Dear Dr. Banerjee,

Thank you for submitting your manuscript to PLOS ONE. After careful consideration, we feel that it has merit but does not fully meet PLOS ONE’s publication criteria as it currently stands. Therefore, we invite you to submit a revised version of the manuscript that addresses the points raised during the review process.

Please submit your revised manuscript by Jul 11 2024 11:59PM. If you will need more time than this to complete your revisions, please reply to this message or contact the journal office at plosone@plos.org. When you're ready to submit your revision, log on to https://www.editorialmanager.com/pone/ and select the 'Submissions Needing Revision' folder to locate your manuscript file.

Please include the following items when submitting your revised manuscript:

- A rebuttal letter that responds to each point raised by the academic editor and reviewer(s). You should upload this letter as a separate file labeled 'Response to Reviewers'.
- A marked-up copy of your manuscript that highlights changes made to the original version. You should upload this as a separate file labeled 'Revised Manuscript with Track Changes'.
- An unmarked version of your revised paper without tracked changes. You should upload this as a separate file labeled 'Manuscript'.

If you would like to make changes to your financial disclosure, please include your updated statement in your cover letter. Guidelines for resubmitting your figure files are available below the reviewer comments at the end of this letter.

If applicable, we recommend that you deposit your laboratory protocols in protocols.io to enhance the reproducibility of your results. Protocols.io assigns your protocol its own identifier (DOI) so that it can be cited independently in the future. For instructions see: https://journals.plos.org/plosone/s/submission-guidelines#loc-laboratory-protocols. Additionally, PLOS ONE offers an option for publishing peer-reviewed Lab Protocol articles, which describe protocols hosted on protocols.io. Read more information on sharing protocols at https://plos.org/protocols?utm_medium=editorial-email&utm_source=authorletters&utm_campaign=protocols.

We look forward to receiving your revised manuscript.

Kind regards,

Gonzalo A. Ruz, Ph.D.

Academic Editor

PLOS ONE

Journal Requirements:

Please review your reference list to ensure that it is complete and correct. If you have cited papers that have been retracted, please include the rationale for doing so in the manuscript text, or remove these references and replace them with relevant current references. Any changes to the reference list should be mentioned in the rebuttal letter that accompanies your revised manuscript. If you need to cite a retracted article, indicate the article’s retracted status in the References list and also include a citation and full reference for the retraction notice.

Additional Editor Comments:

Reviewer 1 points out that some minor aspects still need to be addressed. The authors should work on these points for the paper to be accepted.

[Note: HTML markup is below. Please do not edit.]

Reviewers' comments:

Reviewer's Responses to Questions

**Comments to the Author**

1. If the authors have adequately addressed your comments raised in a previous round of review and you feel that this manuscript is now acceptable for publication, you may indicate that here to bypass the “Comments to the Author” section, enter your conflict of interest statement in the “Confidential to Editor” section, and submit your "Accept" recommendation.

Reviewer #1: All comments have been addressed

**********

2. Is the manuscript technically sound, and do the data support the conclusions?

The manuscript must describe a technically sound piece of scientific research with data that supports the conclusions. Experiments must have been conducted rigorously, with appropriate controls, replication, and sample sizes. The conclusions must be drawn appropriately based on the data presented. 

Reviewer #1: Partly

**********

3. Has the statistical analysis been performed appropriately and rigorously? 

Reviewer #1: Yes

**********

4. Have the authors made all data underlying the findings in their manuscript fully available?

The PLOS Data policy requires authors to make all data underlying the findings described in their manuscript fully available without restriction, with rare exception (please refer to the Data Availability Statement in the manuscript PDF file). The data should be provided as part of the manuscript or its supporting information, or deposited to a public repository. For example, in addition to summary statistics, the data points behind means, medians and variance measures should be available. If there are restrictions on publicly sharing data—e.g. participant privacy or use of data from a third party—those must be specified.

Reviewer #1: Yes

**********

5. Is the manuscript presented in an intelligible fashion and written in standard English?

PLOS ONE does not copyedit accepted manuscripts, so the language in submitted articles must be clear, correct, and unambiguous. Any typographical or grammatical errors should be corrected at revision, so please note any specific errors here.

Reviewer #1: Yes

**********

6. Review Comments to the Author

Please use the space provided to explain your answers to the questions above. You may also include additional comments for the author, including concerns about dual publication, research ethics, or publication ethics. (Please upload your review as an attachment if it exceeds 20,000 characters)

Reviewer #1: While the paper has significantly improved the aspects highlighted in the previous review, there are still some points to improve in order to consider it for publication:

1) Although the resolution of the images has improved considerably, Figure 1 is still difficult to understand. I think the authors should rework this figure to make it clearer.

2) Although it was added in the introduction that: "A drawback of the asynchronous scheme is that the run time for simulations increases rapidly with the number of nodes in the network," no references are provided to support it. It would be interesting to add references from other papers that theoretically or experimentally justify the statement. In addition, regarding the following statement: “Asynchronous update preserved the G0 steady state, but the cyclic attractor became very complex with many intertwined cycles.” The authors should provide more details in what does it mean “cyclic attractor” in the context of stochastic dynamics.

3) I still think that the paper could be greatly improved if it is better situated in the context of the state of the art. How does it compare with other methods?

**********

7. PLOS authors have the option to publish the peer review history of their article (what does this mean?). If published, this will include your full peer review and any attached files.

If you choose “no”, your identity will remain anonymous but your review may still be made public.

**Do you want your identity to be public for this peer review?** For information about this choice, including consent withdrawal, please see our Privacy Policy.

Reviewer #1: No

**********

[NOTE: If reviewer comments were submitted as an attachment file, they will be attached to this email and accessible via the submission site. Please log into your account, locate the manuscript record, and check for the action link "View Attachments". If this link does not appear, there are no attachment files.]

While revising your submission, please upload your figure files to the Preflight Analysis and Conversion Engine (PACE) digital diagnostic tool, https://pacev2.apexcovantage.com/. PACE helps ensure that figures meet PLOS requirements. To use PACE, you must first register as a user. Registration is free. Then, login and navigate to the UPLOAD tab, where you will find detailed instructions on how to use the tool. If you encounter any issues or have any questions when using PACE, please email PLOS at figures@plos.org. Please note that Supporting Information files do not need this step.

# Author comment: Author response to Decision Letter 1

**DOI:** 10.1371/journal.pone.0306523.r004

17 Jun 2024

Please see the file 'Response to Reviewers' in our submission

Attachment Submitted filename: Response to Reviewers.docx

# Editor report: Decision Letter 2

**DOI:** 10.1371/journal.pone.0306523.r005

**Authors:** Gonzalo A. Ruz

20 Jun 2024

A Perturbation Approach for Refining Boolean Models of Cell Cycle Regulation

PONE-D-23-41629R2

Dear Dr. Banerjee,

We’re pleased to inform you that your manuscript has been judged scientifically suitable for publication and will be formally accepted for publication once it meets all outstanding technical requirements.

Within one week, you’ll receive an e-mail detailing the required amendments. When these have been addressed, you’ll receive a formal acceptance letter and your manuscript will be scheduled for publication.

An invoice will be generated when your article is formally accepted. Please note, if your institution has a publishing partnership with PLOS and your article meets the relevant criteria, all or part of your publication costs will be covered. Please make sure your user information is up-to-date by logging into Editorial Manager at Editorial Manager® and clicking the ‘Update My Information' link at the top of the page. If you have any questions relating to publication charges, please contact our Author Billing department directly at authorbilling@plos.org.

If your institution or institutions have a press office, please notify them about your upcoming paper to help maximize its impact. If they’ll be preparing press materials, please inform our press team as soon as possible -- no later than 48 hours after receiving the formal acceptance. Your manuscript will remain under strict press embargo until 2 pm Eastern Time on the date of publication. For more information, please contact onepress@plos.org.

Kind regards,

Gonzalo A. Ruz, Ph.D.

Academic Editor

PLOS ONE

# Editor report: Acceptance letter

**DOI:** 10.1371/journal.pone.0306523.r006

**Authors:** Gonzalo A. Ruz

7 Jul 2024

PONE-D-23-41629R2 

PLOS ONE

Dear Dr. Banerjee, 

I'm pleased to inform you that your manuscript has been deemed suitable for publication in PLOS ONE. Congratulations! Your manuscript is now being handed over to our production team.

At this stage, our production department will prepare your paper for publication. This includes ensuring the following:

* All references, tables, and figures are properly cited

* All relevant supporting information is included in the manuscript submission,

* There are no issues that prevent the paper from being properly typeset

If revisions are needed, the production department will contact you directly to resolve them. If no revisions are needed, you will receive an email when the publication date has been set. At this time, we do not offer pre-publication proofs to authors during production of the accepted work. Please keep in mind that we are working through a large volume of accepted articles, so please give us a few weeks to review your paper and let you know the next and final steps. 

Lastly, if your institution or institutions have a press office, please let them know about your upcoming paper now to help maximize its impact. If they'll be preparing press materials, please inform our press team within the next 48 hours. Your manuscript will remain under strict press embargo until 2 pm Eastern Time on the date of publication. For more information, please contact onepress@plos.org.

If we can help with anything else, please email us at customercare@plos.org.

Thank you for submitting your work to PLOS ONE and supporting open access. 

Kind regards, 

PLOS ONE Editorial Office Staff

on behalf of

Prof. Gonzalo A. Ruz 

Academic Editor

PLOS ONE
